# How far are we from automatic crystal structure solution via molecular-replacement techniques?

**DOI:** 10.1107/S2059798319015468

**Published:** 2020-01-01

**Authors:** Maria Cristina Burla, Benedetta Carrozzini, Giovanni Luca Cascarano, Carmelo Giacovazzo, Giampiero Polidori

**Affiliations:** a Dipartimento di Fisica e Geologia, Università di Perugia, Piazza Università, I-06123 Perugia, Italy; b Istituto di Cristallografia, CNR, Via Amendola 122/O, I-70126 Bari, Italy

**Keywords:** molecular replacement, proteins, nucleic acids, automated model building, structure refinement

## Abstract

Although the success of molecular-replacement techniques requires the solution of a six-dimensional problem, this is often subdivided into two three-dimensional problems. *REMO*09 is one of the programs which have adopted this approach. It has been revisited in the light of a new probabilistic approach which is able to directly derive conditional distribution functions without passing through a previous calculation of the joint probability distributions. The conditional distributions take into account various types of prior information: in the rotation step the prior information may concern a non-oriented model molecule alone or together with one or more located model molecules. The formulae thus obtained are used to derive figures of merit for recognizing the correct orientation in the rotation step and the correct location in the translation step. The phases obtained by this new version of *REMO*09 are used as a starting point for a pipeline which in its first step extends and refines the molecular-replacement phases, and in its second step creates the final electron-density map which is automatically interpreted by *CAB*, an automatic model-building program for proteins and DNA/RNA structures.

## Abstract 2

An automatic pipeline based on molecular-replacement phases is described for the automatic crystal structure solution of protein and DNA/RNA molecules.

## 1. Symbols and abbreviations   

EDM: electron-density modification.

**C**_*s*_ = (**R**_*s*_, **T**_*s*_), with *s* = 1, …, *m*: the symmetry operators of the target structure. **R**_*s*_ is the rotational part, **T**_*s*_ is the translational part and *m* is the number of symmetry operators.

*t*, *t*_*p*_: the numbers of atoms in the asymmetric units of the target and model structure, respectively.

*N* = *mt*, *N_p_* = *mt_p_*: the numbers of atoms in the unit cells of the target structure and model structure, respectively. It is supposed, for the sake of simplicity, that all of the atoms are in general positions. Usually *N*_*p*_ ≤ *N*, but it may also be the case that *N_p_* > *N*.

*f_j_*: the atomic scattering factor of the *j*th atom (thermal factor included).

*F_p_* = 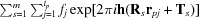 = |*F_p_*|exp(*i*φ_*p*_): structure factor of the model structure. **r**_*pj*_ are the atomic positions of the model structure when it has been well oriented and located.

*F* = 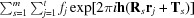 = |*F*|exp(*i*φ): structure factor of the target structure. **r**_*j*_ are the true atomic positions. It is supposed that the target and model molecules are isomorphous, so that **r**_*j*_ = **r**_*pj*_ + Δ**r**_*j*_. Δ**r**_*j*_ is the misfit between the atomic position **r**_*j*_ in the target and the corresponding **r**_*pj*_ in the model structure.

*E* = *A* + *iB* = *R*exp(*i*φ), *E*_*p*_ = *A*_*p*_ + *iB*_*p*_ = *R*_*p*_exp(*i*φ_*p*_): normalized structure factors *F* and *F_p_*, respectively.


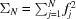
, 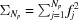: the scattering power at a given sinθ/λ for the target and model structure, respectively.

*D* = 〈cos(2π**h**Δ**r**_*j*_)〉. The average is calculated per resolution shell.

σ_A_ = 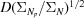. σ_A_ is a statistical estimate of the correlation between the model and target structures (Srinivasan, 1966 ▸). Ideally σ_A_ = 0 for uncorrelated models and σ_A_ = 1 for identical model and target structures.

SI: the sequence identity between model and target molecules.

AMB: automated model building.

## 2. Introduction   

Molecular-replacement (MR) techniques (Rossmann & Blow, 1962 ▸; Rossmann, 1972 ▸, 1990 ▸) aim at phasing an unknown target structure using a known search molecule. The problem to solve is of a six-dimensional nature because it implies the correct orientation and location of the search molecule. Some MR programs face this in six-dimensional space [for example *EPMR* (Kissinger *et al.*, 1999 ▸), *SOMoRe* (Jamrog *et al.*, 2003 ▸) and *Queen Of Spades* (Glykos & Kokkinidis, 2000 ▸); see also Fujinaga & Read (1987 ▸)], even if an exhaustive six-dimensional search is generally avoided. Such programs are, in general, very time-consuming. More frequent is the practice of splitting the MR process into two three-dimensional steps: a rotation and a translation step. The most popular related programs are *X-PLOR*/*CNS* (Brünger, 1992 ▸), *AMoRe* (Navaza, 1994 ▸), *BEAST* (Read, 1999 ▸), *MOLREP* (Vagin & Teplyakov, 2010 ▸) and *Phaser* (McCoy *et al.*, 2007 ▸). In *BEAST* and *Phaser*, maximum-likelihood-based conditional distributions are applied (see Read & McCoy, 2016 ▸, 2018 ▸; McCoy *et al.*, 2018 ▸). Comprehensive reviews of the various techniques (updated up to 2007) have been collected in the January 2008 issue of *Acta Crystallographica Section D*. In recent years, more effort has been dedicated to cases in which the available experimental structures used as search models are only distantly homologous to the target; see, for example, Simpkin *et al.* (2018 ▸), Rigden *et al.* (2018 ▸), Pröpper *et al.* (2014 ▸), Millán *et al.* (2015 ▸) and Cabellero *et al.* (2018 ▸).

In 2009, an MR program (*REMO*09; Caliandro *et al.*, 2009 ▸) was proposed in which a probabilistic approach based on the joint probability distribution method was described. Joint distributions were derived in the absence of or under various prior conditions. For example, in the rotation step the correct rotation of a monomer is found via a figure of merit calculated when other monomers were previously oriented or located, or also when such information is not available. Joint distributions were also derived for the translation step: a monomer is located given its own orientation or the orientations and/or locations of other monomers.

Burla *et al.* (2017 ▸), starting from *REMO*09 phases, checked the efficiency of a phase-refinement pipeline which synergically combines mainstream refinement techniques (specifically *DM*; Cowtan, 2001 ▸) with out-of-mainstream techniques [specifically, *free lunch* (Caliandro *et al.*, 2005*a* ▸,*b* ▸), low-density Fourier transform (Giacovazzo & Siliqi, 1997 ▸), *vive la difference* (Burla, Caliandro *et al.*, 2010 ▸; Burla, Giacovazzo *et al.*, 2010 ▸), *Phantom derivative* (Giacovazzo, 2015*b* ▸; Carrozzini *et al.*, 2016 ▸) and phase-driven model refinement (Giacovazzo, 2015*a* ▸)]. For simplicity, we will refer to this modulus as *SYNERGY*. Burla *et al.* (2017 ▸) automatically submitted the protein data obtained by *SYNERGY* to the AMB procedure *CAB* (Burla *et al.*, 2017 ▸): it applies *Buccaneer* (Cowtan, 2006 ▸) in a cyclic way.

In a recent paper (Giacovazzo, 2019 ▸), the standard method of joint probability distribution functions has been revised and updated. In particular, two-phase, three-phase and four-phase invariants are estimated directly via conditional distributions without passing through a previous calculation of the related joint probability distributions. The probabilistic formulae thus obtained do not coincide, in general, with the corresponding formulae established through the standard study of the joint probability distribution functions. Some of them are immediately applicable to MR, and some others, also suitable for MR, are derived here via this new approach. The formulae thus obtained form the basis for the modified version of *REMO*09 used in this paper.

In this paper, in accordance with the talk given by one of us at the 2019 CCP4 Study Weekend in Nottingham, England, we show the default results obtained on applying the modified *REMO*09 → *SYNERGY* → *CAB* pipeline to a large set of protein and nucleic acid structures. To obtain these results, we extended *CAB* to nucleic acid structures (unpublished work) by making the use of *Nautilus* (Cowtan, 2014 ▸) cyclical. The purposes are twofold: to check the efficiency of the new probabilistic formulae used in the modified version of *REMO*09 and to check how far a modern crystallographic pipeline based on MR phases is from the automatic crystal structure solution of macromolecules.

## 3. General features of *REMO*09   

Various directives allow *REMO*09 users to choose proper approaches for solving macromolecular structures. In this section, we will summarize the default approach used in all of our applications.

(i) The observed and calculated data are scaled by Wilson techniques, which are also used to calculate the normalized structure factors (the observed and calculated 〈*R* ^2^〉 are scaled to unity shell by shell). The isotropic thermal factors of the model atoms are automatically modified to make them compatible with the overall temperature factor of the target structure.
(ii) The target and model sequences are read.
(iii) The orientation space is sampled in terms of Lattman angles (Lattman, 1972 ▸) with an angular step depending on the resolution of the active reflections (the maximum angular step is 5°). The extent of the orientation space is limited to the asymmetric region of the rotation group (Hirshfeld, 1968 ▸). For the first monomer to be located, only the Cheshire cell is explored in the translation step.
(iv) The map grid used in the translation search along each axis is 1/3 of the data resolution for proteins and 1/4 for nucleic acids.
(v) The active reflections for calculating figures of merit used in the rotation and translation searches are automatically selected. Low-resolution reflections (up to 7 Å) are eliminated from the calculations unless the SI is less than 0.5. The highest accepted resolution is 2.5 Å. This limit is extended a little for the translation step owing to the increased prior information gained during the rotation step. The SI is usually less critical for nucleic acids, mostly because nucleic acid helices can adopt similar conformations even when their sequences are drastically different.
(vi) The rotations are ordered according to the rotation figure of merit (RFOM; see Section 4). The good solutions are usually dispersed at the top of the list of ordered solutions: therefore, to speed up calculations only a subset are submitted to the translation step, in which the new figure of merit TFOM is used (see Section 5).

## 4. Rotational search when only one monomer lies in the asymmetric unit of the target structure   

The rotational search is performed by locating the model molecule in a *P*1 cubic unit cell. According to Rabinovich *et al.* (1998 ▸), the structure factors of the model are calculated only once: fitting to the observed data is obtained by rotating the observed reciprocal lattice with respect to the model lattice.

The figure of merit designed for picking up the correct orientation of the model molecule is RFOM, the correlation factor between the observed *R* ^2^ and its expected value 〈*R* ^2^〉 as calculated by the probabilistic approach described by Giacovazzo (2019 ▸). RFOM is expected to be maximum for the correct model orientation and 〈*R* ^2^〉 is the expected value of *R* ^2^ given the prior information on the model stereochemistry:
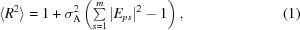
where
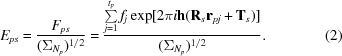

*F_ps_* is the contribution to the calculated model structure factor arising from the asymmetric unit of the model structure, and *E_ps_* is its normalized (with respect to the scattering power of the model structure, symmetry-equivalent molecules included) form. The *E_ps_* are calculated and stored for each reflection via FFT of the electron density of the model structure in the enlarged cubic cell.

(1) has appropriate asymptotic behaviours: *i.e.* when σ_A_ = 0 then 〈*R* ^2^〉 = 1, as it should be in the absence of prior information, and when σ_A_ = 1 then 〈*R* ^2^〉 = 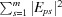. The identity 〈*R* ^2^〉 = *R* ^2^ may only occur in *P*1 when the asymmetric unit contains only one monomer showing a high similarity index to the target molecule.

Despite its good asymptotic properties, the use of (1) did not lead to a very efficient RFOM. The reason may lie in the mathematical definition of σ_A_^2^: according to Carrozzini *et al.* (2013 ▸) it coincides with the correlation factor between |*F*|^2^ and the calculated squared structure factor. In the rotation step the experimental values of σ_A_^2^ are generally small, mostly because 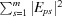 is not the dominant component of the calculated squared structure factor. Thus, in some resolution shells σ_A_ < 0 (anticorrelation situation), while the σ_A_^2^ parameter to be used in (1) remains positive. This suggested that we eliminate the calculation of σ_A_ from (1) and simplify it as
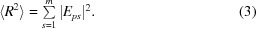
The 200 orientations corresponding to the highest values of RFOM are selected for the translation step: this number is enhanced to 300 if more than one monomer is in the target molecule and to 400 if SI < 0.4.

## 5. Translation search when only one monomer lies in the asymmetric unit of the target structure   

The orientations selected according to Section 4 are submitted to the translation search one by one. This is performed by using the T2 function of Crowther & Blow (1967 ▸) in the form modified by Harada *et al.* (1981 ▸) and by Navaza (1994 ▸). T2 is implemented via FFT, as suggested by Vagin & Teplyakov (1997 ▸).

Only peaks falling inside the Cheshire unit cell are considered. For the same orientation, more peaks can be found: to spare computing time, only the largest five translations per orientation are saved. The selection of the best translations is made via the figure of merit TFOM, coinciding with the correlation factor between the observed amplitude |*F*| and the structure-factor amplitude |*F_p_*| as calculated for each translation.

Some further controls modify the simple approach above.

(i) The translations with the largest TFOM values are submitted to the SIMPLEX method (Rowan, 1990 ▸), an unconstrained optimization technique related to the downhill method (Nelder & Mead, 1965 ▸), which is here applied to a six-dimensional parameter space (three for rotation and three for translation). The method is applied two times to the selected five (or ten for nucleic acids or if SI < 0.4) roto-translations with the largest values of TFOM: they are then submitted to *REFMAC* optimization cycles. The purpose is to optimize the model and better recognize the best solution. The final figure of merit is
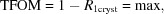
where
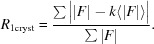

(ii) The clash test (among symmetry-equivalent molecules) is applied, which dumps the TFOM value calculated above when a nonvanishing clash is found. The dumping factor is set to
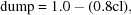
where cl is the percentage of C^α^ atoms in the clash condition. The dumping factor cannot be <0.2.

The roto-translation with the highest figure of merit is automatically submitted to the *SYNERGY* step and to the *CAB* procedure.

## 6. Rotational search when more than one monomer lies in the asymmetric unit of the target molecule   

In the standard *REMO*09 program, when several monomers with the same stereochemistry are present in the asymmetric unit, the following three-step approach is used.

(i) A number of orientations are selected when the orientation of the first monomer is searched.
(ii) Once the first monomer has been located, the orientation of the second monomer is searched among the most probable orientations selected in step (i).
(iii) After the location of the second monomer, steps (i) and (ii) are repeated until all monomers are located.

This simple procedure may not work when the number of monomers in the asymmetric unit is large (more than three) or when the target is constituted of a number of components with different stereochemistry, each contributing a fraction of the scattering power in the asymmetric unit.

This is the case for PDB entries 1lat and 2iff. The first test structure shows two chains of 71 and 74 resideues, respectively, and two identical nucleic acid chains, each with 19 nucleotides. The structure with PDB code 2iff is composed of three protein chains: two with 212 and 214 residues and a third chain with only 129 residues. The model coincides with the third target protein chain.

We then decided to modify the *REMO*09 approach as follows: when the first molecule has been located, the rotations of the second and the others must be searched for using an *ex novo* rotation step and, where the case, by using a different model.

In both of the approaches the figures of merit to be used for recognizing the correct rotation must be designed to take into account that one or more monomers have been previously oriented and located. This increases the signal to noise in the search for the new monomer.

Let us consider the simplest case: the first monomer has been located and we want to orient the second monomer (no other monomers are supposed to lie in the asymmetric unit). Appendix *A* [App appa] suggests that RFOM may still be the correlation factor between the observed *R* ^2^ and its expected value 〈*R* ^2^〉, but now
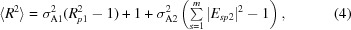
where *R* ^2^_*p*1_ is the squared amplitude of the normalized model structure factor corresponding to the already located first model monomer (normalized with respect to the scattering power of the structure containing the first monomer and its symmetry equivalents) and σ_A1_ is the σ_A_ value corresponding to the pairs (*R*, *R*_*p*1_). The last term on the right-hand side of (4) corresponds to the contribution of the second model monomer (the correct orientation of which we are searching for). σ_A2_ is the σ_A_ value corresponding to the pairs (*R*, 〈*R*_2_^2^〉^1/2^), where
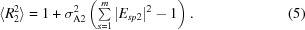
Let us briefly discuss the expected behaviour of (4).

The probabilistic approach used to derive (4) excludes the existence of a mixed nonzero term relating the monomer already positioned to the monomer for which the orientation is searched. Thus, the two contributions are simply additive.

When the first monomer is badly oriented and/or located σ^2^_A1_ is expected to be close to zero. Since σ^2^_A2_ is always expected to be a small value (at least for non-*P*1 space groups; see Section 4), RFOM is expected to be small. When the first monomer is well located and the second is well oriented then RFOM is expected to be larger. However, values of σ^2^_A1_ and σ^2^_A2_ that are both close to unity are not expected because Σ_*p*1_/Σ_*N*_ and Σ_*p*2_/Σ_*N*_ values that are both close to unity are not allowed. Sections 4 and 5 suggest avoiding the use of σ_A_ values so that 〈*R* ^2^〉 reduces to
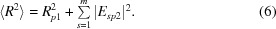
The final RFOM is the correlation coefficient between the observed *R* ^2^ and its expected value 〈*R* ^2^〉. Let us now generalize (6) to the case in which three monomers are contained in the asymmetric unit under the condition that the first and second monomers have already been oriented and located. The expression (6) is still valid; we only have to change the meaning of the symbols. *R*_*p*1_ will represent the normalized amplitude of the model structure corresponding to the first and second monomers (symmetry equivalents included), 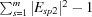 will represent the contribution arising from the monomer for which the correct orientation is searched.

The procedure is now cyclic: the same equation may be applied to any number of monomers.

## 7. Translational search when more than one monomer lies in the asymmetric unit of the target molecule   

Let us first suppose that one monomer has already been oriented and located (*F*_1_ is its generic structure factor) and that a second monomer has been oriented. If we use the Crowther T2 function to locate the second monomer in the translation step then the expected squared structure factor of the structure constituted by the two monomers and their symmetry equivalents in correct positions is
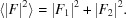
This is a weak relation owing to the fact that 〈|*F*|^2^〉 does not include the mixed term *F*_1_ *F*_2_.

A better approach is that using the translation function involving *F* instead than its square. Let **r**_*pj*_ be the current positional vector of the *j*th atom of the second model monomer: the structure factor of the structure constituted by the second monomer and its symmetry equivalents in correct positions is then
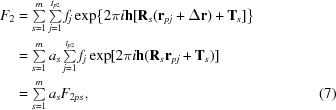
where Δ**r** is a suitable unknown positional shift,
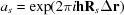
and
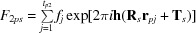
is the component of the current model structure factor.

The algorithm is very simple. *F*_2_ is calculated for each active reflection only once, in the initial position of the second monomer. The second monomer is then moved by the shift Δ**r** on all of the grid points of the asymmetric unit, where *F*_2_ is calculated via (7) and summed with *F*_1_ to obtain
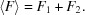



The correct grid position is expected to be that for which TFOM, the correlation factor between the observed amplitude |*F*| and the structure-factor amplitude 〈*F*〉, is a maximum.

The method is simply generalized to locate an *n*th well oriented monomer when the first *n* − 1 monomers have been well oriented and located.

## 8. Applications   

We applied the automatic modified pipeline *REMO*09 → *SYNERGY* → *CAB* to an extended set of test structures, proteins and nucleic acids. We used 80 protein and 38 nucleic acid test structures, the PDB codes of which are reported in Tables 1 ▸ and 2 ▸. The first 34 protein test structures had previously been used by Burla *et al.* (2017 ▸) to check the *SYNERGY* refinement process on standard *REMO*09 phases. Proteins 25–34 belong to the set of 13 structures studied by DiMaio *et al.* (2011 ▸) and characterized by an SI between the model and target structures of lower than 0.30. The experimental data and models for the remaining 46 protein test structures had been deposited in the PDB by the Joint Centre for Structural Genomics, Wilson Laboratory, Scripps Institute: they were used to verify the efficiency of our pipeline on a larger number of test structures (most of them were not originally solved by MR).

The 38 nucleic acid structures were selected from the PDB: we downloaded the observed diffraction data, information on the unit cell, space-group symmetry, published sequences and MR models. 20 of them are DNA and the remaining 18 are RNA fragments. Additional information on all of the test structures is given in Supplementary Tables S1 and S2.

For all of the test structures the same small set of directives was used (coinciding with our default set) such as those shown in Table 3 ▸ for PDB entry 1xyg.

The experimental results are reported in Tables 1 ▸ and 2 ▸. For each test structure PDB is the PDB code, MRP° is the average phase error in degrees at the end of the *REMO*09 step and SYN° is the average phase error in degrees at the end of the *SYNERGY* step. For proteins, MA is the ratio ‘number of C^α^ atoms within 0.6 Å distance from the published positions/number of C^α^ atoms in the asymmetric unit’ as obtained by *CAB*. For nucleic acids, MA is the ratio ‘number of residues with P atoms within 1.3 Å distance from the published positions/number of residues in the asymmetric unit’ in accordance with *CAB* interpretation. We will assume that good models are obtained by *CAB* when MA is sufficiently large: as a rough rule of thumb, we will assume that a good solution has been automatically found when MA > 0.5.

For proteins we observe the following.

(i) Good solutions were found for 64 of the 80 test proteins. The 16 failures are essentially owing to the limited efficiency of *REMO*09. Indeed, for 14 of the 16 failures MRP° was ≥74°: in these conditions *SYNERGY* is often unable to substantially reduce the average phase error so as to allow *CAB* to succeed. *REMO*09 failures are frequent for DiMaio structures because, owing to the extreme low value of SI, the MR step often ends with a large model bias which *SYNERGY* is unable to correct.
(ii) When MRP° is not extremely large, *SYNERGY* dramatically reduces the average phase error. In 15 cases MRP° values in the interval 73–80° are broken down to values of less than 43°, thus allowing *CAB* to succeed.
(iii) *CAB* for proteins is extremely efficient. The MA value is very often close to 100 (a clear signal of successful map interpretation), even in nine of the cases for which *SYNERGY* ended with SYN° > 50°.

The panorama is different for nucleic acids. Such behaviour is in part expected because of the special stereochemistry of DNA/RNA structures. They have a large number of rotatable bonds in the main chain (six, while there are two for proteins); consequently, the conformation at low resolution is often ambiguous (Keating & Pyle, 2012 ▸; Murray *et al.*, 2003 ▸). Our experimental results may be summarized as follows: of the 38 nucleic acid structures only 24 are routinely solved. Ten of the 14 failures may be ascribed to *REMO*09 (*i.e.* for these MRP° ≥ 77°). Four of the remaining five failures are owing to *CAB* failures (*CAB* is unable to interpret the electron-density maps of PDB entries 3tok, 4gsg, 4xqz and 5ihd, for which SYN° ≤ 51°).

*SYNERGY* is again efficient (MPR° values of >70° are broken down to values smaller than 40°).

The above experimental tests indicate that the application of *REMO*09 and *CAB* to DNA/RNA are the weakest points of the pipeline. On the contrary, *SYNERGY*, applied to both nucleic acids and to proteins, and the application of *CAB* to proteins are particularly efficient. The existence of weak points in the pipeline do not allow us to positively answer the question in the title of this paper. There are three simple ways to improve the present situation.

(i) Modify *REMO*09 to give a more modern and efficient version.
(ii) Replace *REMO*09 with a more efficient program.
(iii) Modify the *CAB* algorithms for DNA/RNA structures.

Modifications (i) and (iii) would require supplementary and probably lengthy work which is beyond the purpose of the present paper. For suggestion (ii) the easiest choice would be to replace *REMO*09 by a popular and documented MR tool to check whether the conclusions suggested by the results obtained via our pipeline are confirmed by the inclusion of a better updated MR program. *MOLREP* (Vagin & Teplyakov, 2010 ▸) was our choice: it is also preferred amongst others because of its simple use and its possible automation. Our default *MOLREP* procedure corresponds to the following directives (*i.e.* such as those shown below for PDB entry 1xyg):
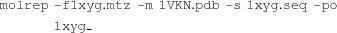



A better default can probably be provided by expert users; therefore, the potential of *MOLREP* is certainly much greater than that corresponding to the naïve default we choose. However, the experimental results obtained by the pipeline *MOLREP* → *SYNERGY* → *CAB*, shown in Tables 4 ▸ and 5 ▸, help to better answer the general question regarding automatic crystal structure solution via MR.

The results in Table 4 ▸ for proteins may be summarized as follows.

(i) Solutions are found for 61 of the 80 test structures. Most of them are owing to our non-optimal *MOLREP* default choice.
(ii) The efficiency of *SYNERGY* and *CAB* is similar to that described for the *REMO*09 → *SYNERGY* → *CAB* pipeline.
(iii) *REMO*09 and *MOLREP* have a complementary behaviour. Indeed, only nine of the 80 protein test structures remained unsolved by both pipelines.

The experimental results in Table 5 ▸ for nucleic acid structures may be summarized as follows.

(i) Of the 38 nucleic acids only 20 are automatically solved: 16 of the 18 failures may be ascribed to the limited effectiveness of our default *MOLREP* procedure (for these MRP° ≥ 86°) and two to *CAB* (PDB entries 3tok, for which SYN° = 47°, and 4gsg, for which SYN° = 55°);
(ii) 14 of the 38 nucleic acid structures remained unsolved by both pipelines.

## 9. Conclusions   

The phase problem for small molecules is considered to be universally solved in practice. The main purpose of this paper is to check whether a similar situation is, or will soon be, available for macromolecules if MR techniques are used. We applied the two pipelines *REMO*09 → *SYNERGY* → *CAB* and *MOLREP* → *SYNERGY* → *CAB* to 80 protein structures and 38 nucleic acid structures. Only nine of the 80 protein structures remained unsolved by both of the pipelines; most of the failures occurred when the SI was extremely low (below 0.30). The increasing availability of better models, the selection of improved default procedures for *REMO*09 and *MOLREP*, and the possible use of more efficient MR programs (*e.g.* *SYNERGY* and *CAB* may use *Phaser*) suggest that automatic crystal structure solution is close for proteins. The situation for nucleic acid structures is different: 14 of the 38 nucleic acid structures remained unsolved by both of the pipelines. Further efforts are therefore necessary to obtain their automatic crystal structure solution: the necessary improvements involve the MR programs (in particular the treatment of ligands, which may be a non-negligible part of the structure) and the AMB section.

## Supplementary Material

Supplementary Tables. DOI: 10.1107/S2059798319015468/ip5004sup1.pdf

## Figures and Tables

**Table 1:** The 80 protein test structures are identified by their PDB codes Their experimental data were submitted to the *REMO*09 + *SYNERGY* + *CAB* pipeline. For each test structure we show MRP°, the average phase error/weighted average phase error in degrees at the end of *REMO*09; SYN°, the average phase error in degrees at the end of the *SYNERGY* step; and MA, the ratio ‘number of C^α^ atoms within 0.6 Å distance from the published positions/number of C^α^ atoms in the asymmetric unit’. Dashes indicate that useful roto-translations were not found by the MR program.

PDB	MRP°	SYN°	MA		PDB	MRP°	SYN°	MA		PDB	MRP°	SYN°	MA
1dy5	55/42	15	99		2f53	58/43	30	95		3nr6	79/67	58	90
1bxo	74/60	28	97		2ayv	54/40	33	89		3zyt	88/89	90	1
2fc3	57/43	32	98		2pby	77/64	36	96		3q6o	80/66	56	99
1tgx	58/44	35	94		2f8m	62/47	41	96		3on5	73/62	43	73
2a46	75/58	31	96		1yxa	74/60	37	95		4fqd	76/61	60	90
1lys	45/36	28	96		2f84	56/42	35	92		3tx8	75/58	47	5
1cgo	78/66	46	100		1cgn	74/64	39	98		3o8s	90/90	89	1
2otb	55/43	34	99		1xyg	64/50	39	98		3npg	79/67	76	3
1kqw	59/46	33	99		2a4k	59/47	32	91		4e2t	74/60	27	96
2sar	54/42	39	96		2b5o	52/40	33	88		3nng	76/61	66	9
1lat	68/55	53	46		1ycn	55/43	31	89					
1e8a	69/54	39	98		2iff	62/53	70	4					

1vkf	90/89	—	—		3mcq	72/57	47	94		4mru	76/67	73	23
1vki	73/56	37	100		3mdo	56/41	31	96		4ogz	68/54	47	96
1vl2	90/90	—	—		3mz2	89/90	—	—		4ouq	49/36	29	98
1vl7	71/57	42	95		3nyy	77/68	50	96		4q1v	72/60	44	98
1vlc	69/55	31	95		3obi	89/90	—	—		4q34	70/53	36	99
2wu6	55/43	38	97		3oz2	74/62	37	93		4q53	62/49	32	95
2x7h	67/59	51	98		3p94	61/46	38	97		4q6k	64/48	34	99
3e49	75/61	52	97		3ufi	77/65	38	94		4q9a	81/76	89	1
3gp0	75/61	40	96		3us5	66/52	37	98		4qjr	66/51	35	88
3h9e	56/43	34	97		4e2e	54/40	39	89		4qni	74/63	42	82
3h9r	63/48	50	87		4ef2	69/52	38	96		4r0k	53/39	33	99
3khu	90/90	—	—		4ezg	68/50	28	98		4rvo	74/61	69	8
3l23	73/56	41	94		4fvs	89/88	—	—		4rwv	69/54	39	94
3llx	69/55	33	99		4gbs	55/38	36	85		4yod	71/56	68	99
3m7a	76/61	41	98		4gcm	65/50	32	98					
3mbj	75/59	43	97		4ler	69/50	30	98					

**Table 2:** The 38 nucleic acid test structures are identified by their PDB codes Their experimental data were submitted to the *REMO*09 + *SYNERGY* + *CAB* pipeline. For each test structure we give MRP°, the average phase error/weighted average phase error in degrees at the end of *REMO*09; SYN°, the average phase error in degrees at the end of the *SYNERGY* step; and MA, the ratio ‘number of residues with P atoms within 1.3 Å distance from the published positions/number of residues in the asymmetric unit’. Dashes indicate that useful roto-translations were not found by the MR program.

PDB	MRP°	SYN°	MA		PDB	MRP°	SYN°	MA		PDB	MRP°	SYN°	MA
1iha	38/27	41	88		4enc	37/27	28	87		5l4o	68/54	41	83
1q96	90/90	—	—		4gsg	54/41	51	25		5lj4	42/30	32	77
1z7f	39/27	35	100		4ms5	70/57	60	78		5mvt	65/55	29	95
2a0p	34/24	32	100		4wo3	88/88	—	—		5nt5	28/18	26	100
2b1d	83/81	82	2		4xqz	53/37	48	2		5nz6	42/29	27	93
2fd0	49/36	33	95		4zym	77/68	80	0		5t4w	46/33	27	100
2pn4	47/34	42	61		5cv2	89/90	—	—		5tgp	72/56	34	86
3ce5	60/51	48	57		5dwx	75/63	63	59		5ua3	84/80	85	0
3d2v	77/69	60	27		5fj0	89/88	—	—		5ux3	90/90	—	—
3eil	62/47	47	79		5i4s	51/40	38	64		5uz6	73/64	34	99
3fs0	68/51	34	100		5ihd	70/51	39	13		5zeg	88/89	—	—
3n4o	36/26	35	73		5ju4	50/33	27	95		6az4	56/42	43	90
3tok	60/45	49	14		5kvj	65/50	51	94					

**Table 3:** Directives for the default use of the *REMO*09/*SYNERGY*/*CAB* pipeline The example refers to the protein with PDB code 1xyg.

%cab buccaneer
%structure 1xyg
%job Molecular Replacement Test on 1xyg
%data
mtz 1xyg.mtz
label H K L F SIGF
sequence 1xyg.seq
%remo
fragment 1vkn.pdb
%end

**Table 4:** The 80 protein test structures are identified by their PDB codes Their experimental data were submitted to the *MOLREP* + *SYNERGY* + *CAB* pipeline. For each test structure we give MRP°, the average phase error/weighted average phase error in degrees at the end of *MOLREP*; SYN°, the average phase error at the end of the SYNERGY step; and MA, the ratio ‘number of C^α^ atoms within 0.6 Å distance from the published positions/number of C^α^ atoms in the asymmetric unit’. Dashes indicate that useful roto-translations were not found by the MR program.

PDB	MRP°	SYN°	MA		PDB	MRP°	SYN°	MA		PDB	MRP°	SYN°	MA
1dy5	90/90	—	—		2f53	66/58	71	8		3nr6	86/83	83	1
1bxo	76/68	29	98		2ayv	56/46	31	94		3zyt	90/91	—	—
2fc3	57/44	32	98		2pby	70/62	33	97		3q6o	83/79	78	9
1tgx	61/49	35	94		2f8m	65/55	37	99		3on5	89/89	89	1
2a46	69/59	29	98		1yxa	76/69	36	95		4fqd	83/79	81	3
1lys	68/62	50	96		2f84	58/47	32	94		3tx8	—	—	—
1cgo	—	—	—		1cgn	77/69	35	100		3o8s	90/90	—	—
2otb	—	—	—		1xyg	63/53	35	94		3npg	89/89	—	—
1kqw	62/52	32	98		2a4k	62/53	30	93		4e2t	79/72	31	96
2sar	53/41	39	95		2b5o	52/41	31	88		3nng	78/70	66	19
1lat	89/89	—	—		1ycn	58/47	30	90					
1e8a	71/62	35	98		2iff	67/60	69	3					

1vkf	84/76	51	96		3mcq	82/73	49	93		4mru	69/60	45	98
1vki	81/73	35	100		3mdo	52/40	31	97		4ogz	68/58	47	96
1vl2	77/68	42	97		3mz2	90/90	—	—		4ouq	52/42	29	99
1vl7	77/69	63	92		3nyy	83/79	76	14		4q1v	72/64	44	97
1vlc	67/56	47	71		3obi	80/74	44	97		4q34	77/67	37	99
2wu6	59/50	38	97		3oz2	79/72	37	94		4q53	64/55	33	96
2x7h	49/40	38	98		3p94	58/48	37	97		4q6k	53/42	35	99
3e49	63/51	45	96		3ufi	78/71	39	91		4q9a	71/61	45	97
3gp0	74/67	42	97		3us5	67/56	37	98		4qjr	67/55	36	82
3h9e	59/47	32	98		4e2e	55/45	39	94		4qni	78/70	42	81
3h9r	73/65	68	2		4ef2	73/63	38	98		4r0k	44/34	30	99
3khu	77/69	56	93		4ezg	79/67	27	98		4rvo	78/70	67	32
3l23	75/65	41	96		4fvs	74/65	60	86		4rwv	70/59	39	93
3llx	74/64	34	99		4gbs	57/43	37	89		4yod	71/61	70	89
3m7a	76/68	41	99		4gcm	65/52	32	98					
3mbj	77/69	43	95		4ler	78/70	63	65					

**Table 5:** The 38 nucleic acid test structures are identified by their PDB codes Their experimental data were submitted to the *MOLREP* + *SYNERGY* + *CAB* pipeline. For each test structure we give MRP°, the average phase error/weighted average phase error in degrees at the end of *MOLREP*; SYN°, the average phase error in degrees at the end of the SYNERGY step; and MA, the ratio ‘number of residues with P atoms within 1.3 Å distance from the published positions/number of residues in the asymmetric unit’. Dashes indicate that useful roto-translations were not found by the MR program.

PDB	MRP°	SYN°	MA		PDB	MRP°	SYN°	MA		PDB	MRP°	SYN°	MA
1iha	71/61	28	94		4enc	52/41	28	88		5l4o	74/64	37	86
1q96	90/89	—	—		4gsg	59/52	55	6		5lj4	67/55	30	95
1z7f	49/36	27	100		4ms5	88/87	—	—		5mvt	68/55	24	100
2a0p	40/31	32	100		4wo3	87/87	—	—		5nt5	51/37	25	100
2b1d	87/86	—	—		4xqz	88/89	—	—		5nz6	44/34	25	88
2fd0	61/52	25	100		4zym	87/87	—	—		5t4w	61/47	27	91
2pn4	49/37	39	64		5cv2	88/90	—	—		5tgp	77/71	49	86
3ce5	72/68	58	57		5dwx	87/86	—	—		5ua3	86/83	—	—
3d2v	90/90	—	—		5fj0	—	—	—		5ux3	89/87	—	—
3eil	85/82	83	23		5i4s	67/63	38	82		5uz6	72/62	65	93
3fs0	74/66	33	100		5ihd	88/89	—	—		5zeg	88/89	—	—
3n4o	43/26	30	85		5ju4	88/89	—	—		6az4	57/45	43	95
3tok	67/54	47	17		5kvj	59/52	54	91					

## Appendix A On the orientation of a second monomer

The problem that we will treat in this appendix is the following: if the first monomer has been correctly oriented and located, how do we fix the orientation of a second monomer? To answer this question, in the following probabilistic approach we will explicitly consider the case in which the orientation of the second monomer has been fixed while its location is unknown. We will see that the conclusive formulae thus obtained may be applied to fix the orientation of the second monomer.

Let *t*_1_ and *t*_*p*1_ be the number of non-H atoms of the first target monomer and of its model molecule, respectively: for simplicity, we are supposing that *t*_1_ ≥ *t*_*p*1_. *t*_2_ and *t*_*p*2_ are the equivalent numbers for the second target monomer and for its model molecule. We order the atoms in the target asymmetric unit so that its structure factor may be represented as

where *t* = *t*_1_ + *t*_2_ is the number of non-H atoms in the target asymmetric unit. In our probabilistic approach **h** is fixed while the positional vectors are the primitive random variables. **U** is an overall free translation vector that is necessary to locate the second monomer in the correct position and Δ**r**_*j*_ are local variables relating the atomic positions of the target monomers to the corresponding positions of the model. In order, (8) may be rewritten as

The atoms contributing to *F*_1_ are related to the atoms of the model molecule of the first monomer via the local shift vectors Δ**r**_*j*_ only (the first monomer has been already located). The atoms contributing to *F*_2_ are related to the atoms of the model molecule of the second monomer through the local shift vectors Δ**r**_*j*_ and through the unknown overall translation vector **U** (indeed, the second monomer has not been located). The coordinates of the atoms contributing to *F*_*q*1_ and *F*_*q*2_ are not related to the atoms of the model molecules; they may be thought of as unconstrained unknown variables.

We now calculate the average value of |*F*|^2^ given the prior information described above,

The above equation may be more explicitly written if the cases in which *i* = *j* and/or *s*1 = *s*2 are emphasized. We have

where *D*_1_ and *D*_2_ are the *D* values (see Section 1) calculated for monomers 1 and 2, respectively. Let us now take into account the relations (11), (12) and (14) below.

so that

*F*_*p*1_ is the structure factor corresponding to the structure constituted of the model molecule that has already been located (and its symmetry equivalents).

where

is the contribution to the structure factor of the model molecule of the second monomer (oriented but not located) arising from the asymmetric unit. In accordance with (14), we have

Substituting (11), (13) and (15) into (10) gives

Dividing the left- and right-hand sides of (16) by Σ_*N*_ leads to

from which

where

*R* ^2^ is normalized with respect to the scattering power of the full target unit cell, *R* ^2^_*p*1_ is normalized with respect to the scattering power of the structure constituted of the oriented and located molecule (symmetry equivalents included) and |*E*_*ps*2_|^2^ is normalized with respect to the scattering power of the model molecules (symmetry equivalents included) that are oriented but not located.

We can now return to the question: why did we formulate a probabilistic theory for the case in which one monomer is well located and the second well oriented, when we are primarily interested in the case in which one monomer is well located and we are looking for the orientation of the second monomer? The answer is simple. Indeed, when we continuously rotate reciprocal space and look for the best fit between *R* ^2^ and 〈*R* ^2^〉 we hope to find a rotation in which the second monomer is well oriented. In this case 〈*R* ^2^〉 will really be the expected value of *R* ^2^ in accordance with (17), while for all of the other orientations this condition will not be obeyed. Accordingly, the correlation will be a maximum.
