# Supplementary material for: How far are we from automatic crystal structure solution via molecular-replacement techniques?
Source: Acta Crystallogr D Struct Biol. 2020 Jan 1;76(Pt 1):9–18. doi: 10.1107/S2059798319015468 (PMC6939436; doi:10.1107/S2059798319015468)
Supplement: Supplementary file 1 [file d-76-00009-sup1.pdf]

STRUCTURAL
BIOLOGY

Volume 75 (2019)

Supporting information for article:

How far are we from automatic crystal structure solution *via* molecular-replacement techniques?

Maria Cristina Burla, Benedetta Carrozzini, Giovanni Luca Cascarano, Carmelo Giacovazzo and Giampiero Polidori

Table S1 80 protein test structures are identified by their PDB code.

For each test structure we give:

Sequence Identity (SI)

Coverage (COV): it is the ratio "number of residues matching the known sequence/ number of residues in the sequence".

RMSD on C-alpha atoms between search model and target (in Å)

Number of molecules (nM) per asymmetric unit. A double number characterizes chains which are different.

PDB	SI	RMSD	nM	COV	PDB	SI	RMSD	nM	COV	PDB	SI	RMSD	nM	COV
1dy5	0.99	0.272	2	100	2f53	0.98	1.031	1	96	3nr6	0.28	2.085	2	88
1bxo	0.54	1.146	1	100	2ayv	0.79	0.840	1	86	3zyt	0.23	2.120	1	59
2fc3	0.60	0.816	1	100	2pby	0.43	1.485	4	98	3q6o	0.15	1.945	1	100
1tgx	0.83	0.649	3	100	2f8m	0.40	1.205	2	97	3on5	0.24	2.579	2	87
2a46	0.41	0.947	1	95	1yxa	0.45	1.683	2	95	4fqd	0.28	1.895	2	93
1lys	0.95	0.605	2	100	2f84	0.65	0.996	1	100	3tx8	0.24	2.243	1	98
1cgo	0.34	1.743	1	100	1cgn	0.35	1.740	1	100	3o8s	0.22	1.508	1	54
2otb	0.65	0.505	2	100	1xyg	0.46		4	96	3npg	0.21	2.400	4	66
1kqw	0.74	0.867	1	100	2a4k	0.59	0.926	2	89	4e2t	0.99	1.223	2	95
2sar	1.00	0.242	2	99	2b5o	0.63	1.164	2	73	3nng	0.25	1.830	2	---
1lat	0.90	0.936	2+2	100	1ycn	0.72	1.164	2	96					
1e8a	0.38	1.520	2	98	2iff *	0.23	0.498	1+1+1	---					

PDB	SI	RMSD	nM	COV	PDB	SI	RMSD	nM	COV	PDB	SI	RMSD	nM	COV
1vkf	0.96	1.544	4	---	3mcq	0.96	1.433	1	97	4mru	0.97	1.177	2	60
1vki	0.99	1.287	2	99	3mdo	1.00	0.660	2	94	4ogz	0.93	1.218	2	97
1vl2	0.99	1.027	4	---	3mz2	0.92	2.105	2	---	4ouq	0.96	0.474	1	100
1vl7	0.95	1.048	2	100	3nyy	0.91	1.483	1	100	4q1v	0.92	1.418	2	100
1vlc	0.98	1.379	1	100	3obi	0.98	1.795	4	---	4q34	0.89	1.436	1	100
2wu6	1.00	0.447	1	89	3oz2	0.97	1.535	1	90	4q53	0.98	0.795	2	99
2x7h	0.98	1.400	2	93	3p94	0.99	0.909	4	100	4q6k	1.00	0.790	4	100
3e49	1.00	0.587	4	98	3ufi	0.89	2.096	1	91	4q9a	0.97	1.695	2	60
3gp0	0.95	1.721	1	97	3us5	0.94	0.900	1	99	4qjr	0.98	1.459	2	94
3h9e	1.00	0.767	2	99	4e2e	0.99	0.994	2	99	4qni	0.91	1.686	1	94
3h9r	1.00	1.149	2	87	4ef2	0.98	1.032	1	100	4r0k	1.00	0.460	2	99
3khu	0.96	1.551	6	---	4ezg	0.97	0.623	2	100	4rvo	0.99	1.286	4	67
3l23	0.92	1.320	1	92	4fvs	0.99	1.260	6	---	4rwv	0.94	1.241	2	97
3llx	1.00	0.968	1	99	4gbs	0.94	0.908	2	100	4yod	1.00	0.824	1	97
3m7a	1.00	1.052	2	100	4gcm	1.00	0.790	2	100	3mbj	0.98	1.458	1	100
4ler	0.97	0.970	1	98										

Table S2 37 nucleic acid test structures are identified *via* their PDB code

For each test structure we give the number of molecules (nM) per asymmetric unit and number of nucleotides in each molecule (nN).

PDB	nM	nN	PDB	nM	nN	PDB	nM	nN
1iha	2	9	4enc	1	52	5l4o	1	77
1q96	3	27	4gsg	2+2	(10;10)	5lj4	2	12
1z7f	3	16	4ms5	1	10	5mvt	2	12
2a0p	2	8	4wo3	2	22	5nt5	2	12
2b1d	6	12	4xqz	8	6	5nz6	3+3	(25;8)
2fd0	2	23	4zym	4	11	5t4w	2	12
2pn4	2+2	(24;20)	5cv2	2	14	5tgp	2	8
3ce5	2	12	5dwx	1+1	(24;8)	5ua3	2	20
3d2v	2	77	5fj0	3	19	5ux3	1+1	(25;8)
3eil	6	12	5i4s	2	12	5uz6	3+3	(25;8)
3fs0	1+1	(10;11)	5ihd	4	6	5zeg	3	23
3n4o	2	12	5ju4	2	12	6az4	1+1	(32;9)
3tok	1+1	(10;10)	5kvj	1+1	(16;16)			
